# Intraosseous Solitary Fibrous Tumor of the Maxilla: A Report of an Unusual Case

**DOI:** 10.7759/cureus.103820

**Published:** 2026-02-18

**Authors:** Yohana Corredor, Carlos Manresa, Miguel Flores, Aubert Brito, Mariana Villarroel-Dorrego

**Affiliations:** 1 Department of Oral and Maxillofacial Surgery, Hospital General del Oeste Dr. José Gregorio Hernández, Caracas, VEN; 2 Dental Research Institute, Universidad Central de Venezuela, Caracas, VEN

**Keywords:** cd34 expression, intraosseous tumor, maxillary tumor, solitary fibrous tumor, stat6

## Abstract

Solitary fibrous tumor (SFT) is a mesenchymal fibroblastic neoplasm initially described in the pleura and subsequently reported in other anatomical sites, primarily affecting the extremities and deep soft tissues. It is driven by the NAB2::STAT6 fusion and presents with nonspecific clinical features, necessitating thorough histopathological evaluation and the use of an immunohistochemical panel for diagnosis. SFT is extremely rare in the maxillofacial region and even more uncommon when involving bone. This case report describes a 31-year-old male who presented with a slow-growing, progressive, painless, deforming mass in the left posterior maxillary region, with 14 months of evolution. After clinical and imaging evaluation, an incisional biopsy was performed. Histopathological and immunohistochemical studies confirmed SFT. Surgical excision of the lesion with preservation of oncologic margins was performed, achieving complete tumor removal. This case highlights an unusual presentation of SFT in the maxilla of a young patient. Diagnosis requires correlation of clinical, imaging, and histopathological findings, with immunohistochemistry using specific markers being essential. Complete surgical resection remains the treatment of choice, and prolonged follow-up is recommended due to the risk of local recurrence, contributing to the understanding of this rare entity in the maxillofacial region.

## Introduction

Solitary fibrous tumor (SFT) is an uncommon mesenchymal neoplasm of fibroblastic origin, first described by Klemperer and Coleman in 1931 [1-4]. Although initially considered a pleural pathology, it is now recognized as a distinct entity capable of arising in various extrapleural sites, including the meninges, peritoneum, abdominal cavity, trunk, extremities, and maxillofacial region [5-7]. Its occurrence in the maxilla is exceptional and poses significant clinicopathological challenges. These challenges arise from the wide anatomic variability of affected regions, such as the buccal mucosa [5,8-11], tongue [5,10], palate [4,5,8-10,12,13], and floor of the mouth [5], as well as the histologic heterogeneity and broad range of differential diagnoses associated with the oral cavity and maxillary bone [5,7,10,14].

Robinson et al. [15] provided fundamental insights into the molecular biology of SFT by identifying the NAB2-STAT6 gene fusion as its defining genetic hallmark. This molecular alteration leads to intense nuclear expression of STAT6, detectable via immunohistochemistry, which has markedly improved diagnostic accuracy to rates exceeding 90% [16-18]. The NAB2-STAT6 fusion arises from a recurrent inversion on chromosome 12 and results in constitutive activation of early growth response genes, driving tumor growth. Its discovery has revolutionized diagnosis, allowing nuclear STAT6 immunohistochemistry to serve as a highly sensitive and specific surrogate for the molecular fusion itself [15-18].

Clinically, maxillary SFTs typically present as slow-growing masses that may occasionally cause local bone destruction [10,14,19]. CT and MRI are essential for determining tumor extent and guiding surgical planning [5,10,20]. The gold standard of treatment remains complete surgical excision with negative margins [11,14]. Given that maxillofacial involvement is rare and intraosseous presentations are even more infrequent, we report a case of an intraosseous maxillary lesion successfully diagnosed and treated as an SFT.

## Case presentation

A 31-year-old male presented to the Oral and Maxillofacial Surgery Service at Hospital General del Oeste Dr. José Gregorio Hernández, Caracas, Venezuela, with a slow-growing, progressive, painless, indurated tumoral lesion in the maxillary region that had developed over 14 months.

The patient reported an unremarkable medical history with no prior surgeries or chronic medication use. His chief complaint was a progressive increase in palatal volume associated with grade III mobility of the posterior molars. Clinical findings were limited to masticatory impairment, with no sinonasal symptoms, facial pain, or halitosis.

**Figure 1:**
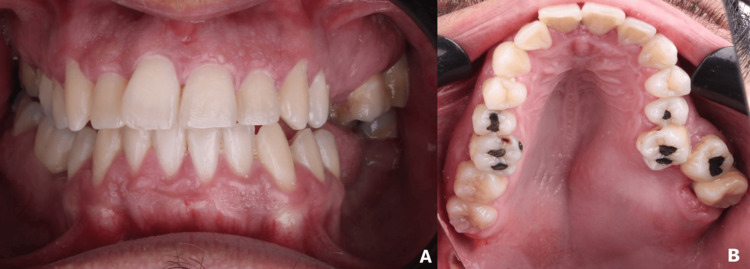
Initial intraoral examination (A) Initial intraoral photograph, (B) with occlusal view, showing a tumoral lesion of the left palate causing displacement of teeth #27 and #28.

On clinical examination, a firm swelling was observed in the posterior left maxillary region, extending from the vestibule to the midline of the palate, covered by mucosa of normal appearance. The molars in the area appeared displaced and showed marked mobility. There was no extraoral deformity, facial asymmetry, or regional lymphadenopathy (Figure 1).

**Figure 2:**
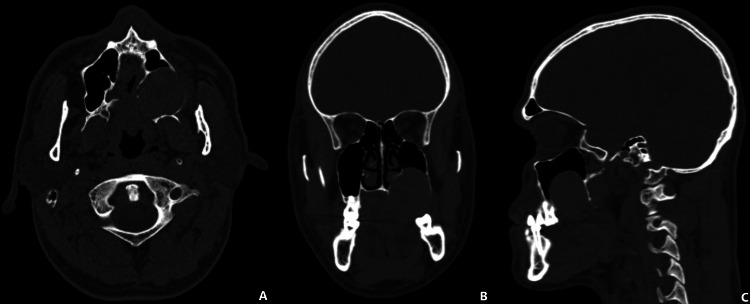
CT of the facial skeleton (A) Axial section showing extension of the lesion to the palatal midline. (B) Coronal section demonstrating expansion of the lateral wall of the maxillary sinus and extension toward the nasal fossa. (C) Sagittal section showing the posterior limit of the lesion at the pterygoid process.

A CT scan revealed an isodense, rounded lesion measuring 3.1 × 3.29 cm within the left maxillary bone. The mass occupied the alveolar process and part of the maxillary sinus, extending toward the inferior portion of the ipsilateral nasal fossa (Figure 2).

**Figure 3:**
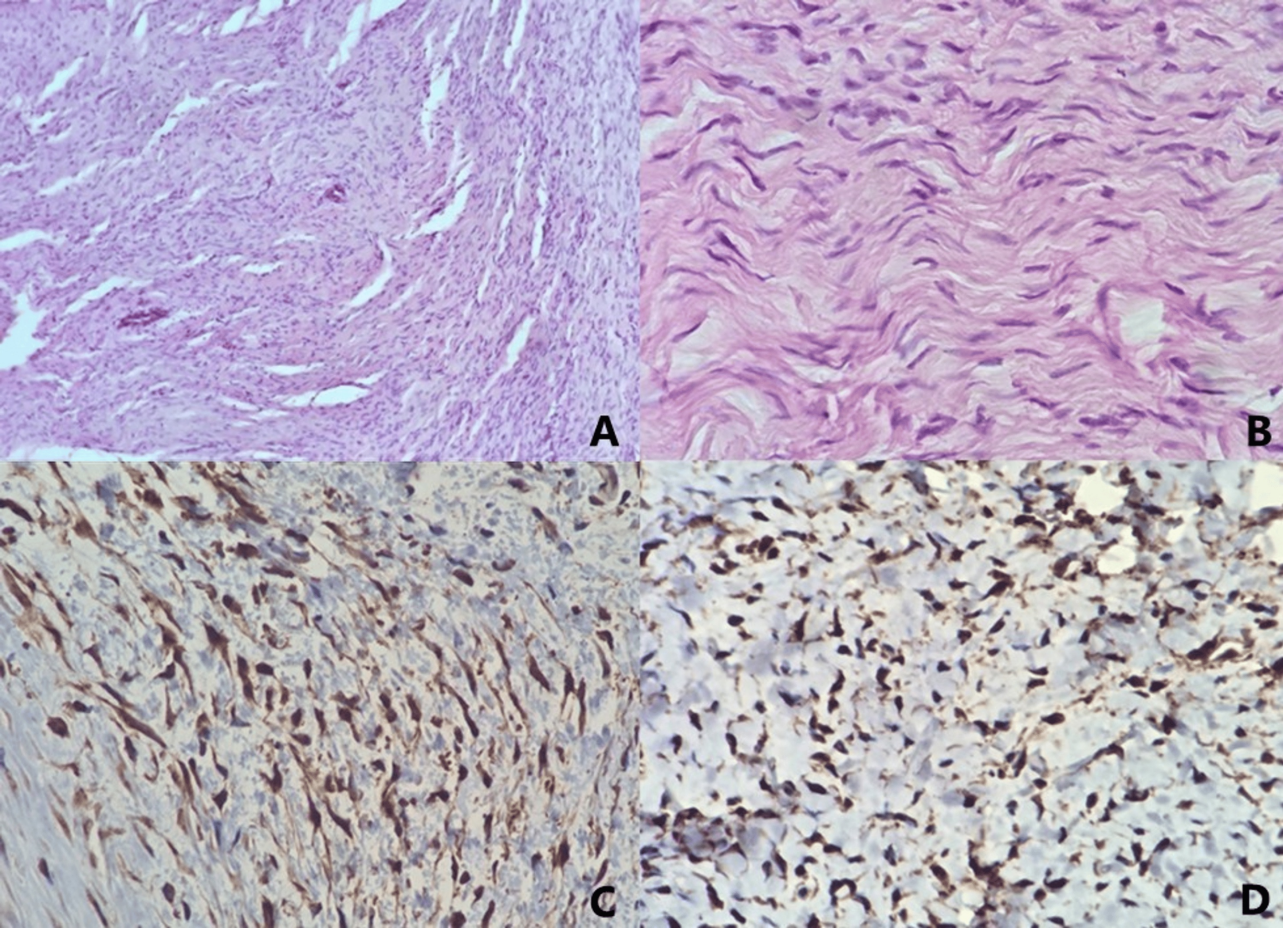
Histopathology study (A) Microphotographs of a benign, well-circumscribed neoplasm composed of numerous spindle cells arranged in an interlacing fascicular pattern (4× H&E). (B) Mitoses are scant (<1 per 2 mm²). Nuclei are small, some with a wavy shape. No atypia or tissue necrosis was observed. Peripheral zones show abundant collagen (10× H&E). (C, D) Strong nuclear expression of STAT6 is shown.

An incisional biopsy was performed under local anesthesia. Histopathological examination revealed a neoplasm composed of spindle-shaped cells with small, uniform nuclei, without atypia or mitotic activity. The definitive diagnosis of SFT was confirmed by immunohistochemistry, which was positive for STAT6 and negative for SMA, S100 protein, SOX10, CD68, desmin, MUC-4, and, unexpectedly, CD34 (Figure 3), with STAT6 serving as the confirmatory diagnostic marker.

**Figure 4:**
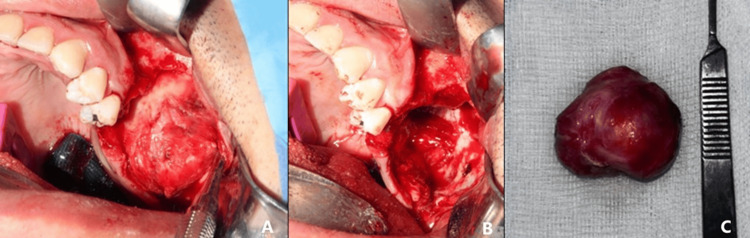
Surgical approach for complete tumor removal (A) Tumoral lesion in the maxilla. (B) Surgical bed after excision of the lesion. (C) Macroscopic view of the specimen submitted for histopathologic and immunohistochemical study.

Subsequently, the patient was taken to the operating room under general anesthesia. A trapezoidal flap was raised, and teeth #26 and #27 were extracted. Subperiosteal dissection was carried down to the lesion, which had well-defined margins and no significant adhesions to surrounding structures, allowing complete excision without complications. A peripheral osteotomy and irrigation of the left maxillary sinus were then performed. Hemostasis was achieved, and the surgical site was closed in layers using absorbable sutures (Figure 4).

The entire specimen was submitted for analysis, and the diagnosis was confirmed by diffuse STAT6 expression throughout the lesion. Risk stratification was performed using a four-tier model based on mitotic count, pleomorphism, and cellularity. Additionally, the French Sarcoma Group risk calculators were applied to estimate overall survival, local recurrence, and distant metastatic risk by integrating clinical data (age and tumor site), pathological features, and radiotherapy history (none) [21].

**Figure 5:**
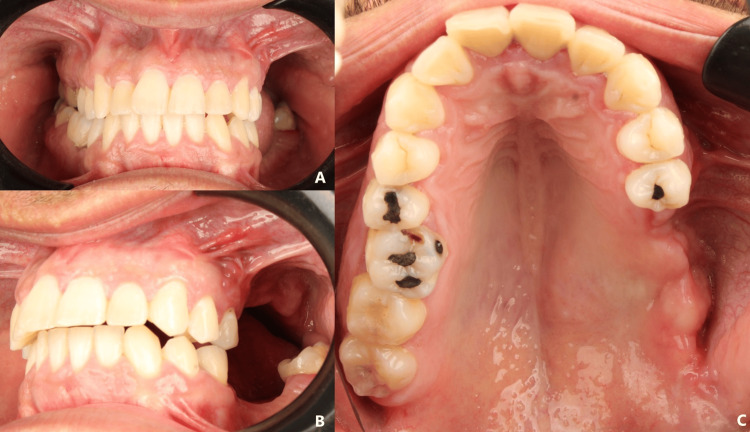
Four-month postoperative follow-up (A) Clinical intraoral photograph, frontal view. (B) Clinical intraoral photograph, lateral view. (C) Clinical intraoral photograph, occlusal view showing the postoperative scar and palate without a lesion.

At four months postoperatively, the soft tissues demonstrated adequate healing with no evidence of oroantral communication. There were no signs of recurrence, and the patient remains under close postoperative surveillance. Prosthetic rehabilitation is currently being planned (Figure 5).

## Discussion

SFT is a fibroblastic neoplasm characterized by NAB2-STAT6 gene rearrangement [1-4]. Previous studies indicate that SFTs of the head and neck region account for approximately 6% of all reported cases [10]. Some reports, including Wang et al. [5] and Pourfarrokh et al. [8], suggest a slight female predominance. Age distribution is typically between the fifth and seventh decades of life [5,10], making the present case, involving a 31-year-old male, outside these typical demographics.

The clinical presentation of SFT is related to the mass effect of the tumor. Tumors in the head and neck often become symptomatic earlier than those in the pleura or abdomen, allowing earlier diagnosis [20]. de Morais et al. [10] describe SFT as a painless, slow-growing nodular mass that may occasionally cause pain due to compression of adjacent structures. Depending on location, the tumor can produce facial asymmetry [14], which Wang et al. [5] attribute to tumor size, as slow growth may go unnoticed. Nunes et al. [7] note that pain is rarely reported, though ulcers may form following local trauma. In the present case, the tumor caused palatal deformity and dental displacement, but no mucosal changes or symptoms were observed.

Regarding anatomical location, Nunes et al. [7] report the buccal mucosa as the most frequently affected site, followed by the tongue and palate, whereas Pourfarrokh et al. [8] identify the tongue as the most common site. Other locations described include the floor of the mouth, submandibular space, and sublingual gland [5], as well as the maxillary sinus, orbital, and nasal cavities, sometimes deforming the palate [20], which parallels the presentation in this case.

Histopathologically, most SFTs exhibit spindle tumor cells arranged randomly with abundant “staghorn” vasculature and alternating hypo- and hypercellular areas [7,9,12]. Hypercellular regions display disorganized spindle cells with minimal stroma [1,5,10]. These features were observed in the incisional biopsy of our patient, but they are insufficient for definitive diagnosis due to histologic heterogeneity and overlap with other fibroblastic neoplasms or low-grade sarcomas [1,5]. de Morais et al. [10] note that differential diagnosis should include fibroblastic lesions with abundant collagen and vascular tumors.

SFT must be distinguished from neurofibroma, schwannoma, myofibroma, leiomyoma, and odontogenic neoplasms [10]. Benign mesenchymal tumors of the maxillofacial region can mimic SFT clinically and radiologically. Schwannomas show strong S-100 positivity, and leiomyomas express muscle markers such as SMA and desmin; SFTs are typically negative for these markers and positive for CD34, CD99, Bcl-2, and, most importantly, STAT6 [2,3]. For intraosseous maxillary lesions, the differential includes odontogenic tumors with mesenchymal components (e.g., odontogenic myxoma, odontogenic fibroma, and fibro-osseous lesions), which may present as expansile radiolucencies similar to SFT but lack NAB2-STAT6 fusion and nuclear STAT6 expression [3,4]. Vascular lesions such as juvenile angiofibroma or intraosseous hemangiomas may also be considered, but these express endothelial markers (CD31 and ERG) and do not show nuclear STAT6 positivity, allowing clear differentiation [2,5].

Accurate diagnosis of spindle cell neoplasms, including spindle squamous cell carcinoma and synovial sarcoma, requires integration of clinical, radiologic, histopathologic, and immunohistochemical findings. Spindle cell carcinoma may show squamous areas and is positive for cytokeratin and vimentin. Synovial sarcoma requires a broader panel, with TLE-1 considered key for diagnosis. Nuclear STAT6 expression and identification of the NAB2-STAT6 fusion are essential for excluding other spindle cell tumors in the maxillofacial region [3,4]. In this case, nuclear STAT6 positivity confirmed a diagnosis of low-grade SFT, consistent with recent literature establishing STAT6 as the standard marker to distinguish SFT from other neoplasms [20].

While most SFTs follow an indolent course, approximately 10% exhibit aggressive behavior, leading to late local or systemic recurrence [21]. Prognostication has evolved from a simple benign/malignant classification to a comprehensive multivariate scoring. Key frameworks include a four-tier histologic model assessing mitoses, pleomorphism, and cellularity, and the French Sarcoma Group calculators, which integrate clinical factors (age and tumor site), pathological features, and previous radiotherapy. Metastatic risk is commonly stratified into low, intermediate, and high categories based on age (≥55 years), tumor size, and a mitotic threshold of ≥2 mitoses/mm² [21].

The treatment of choice is complete surgical excision with negative margins, which has demonstrated low recurrence rates in low-grade tumors [6,9,13]. In the present case, the surgical approach allowed complete removal with clear margins, suggesting a favorable prognosis. Nevertheless, prolonged follow-up is recommended, as local recurrence can occur years after surgery, even in low-grade tumors [4,12,15].

## Conclusions

Intraosseous SFT of the maxilla is extremely rare and presents a significant diagnostic challenge. This case highlights that SFT should be considered in the differential diagnosis of atypical maxillofacial neoplasms. Definitive diagnosis requires integration of clinical, radiologic, and histopathologic findings, with STAT6 immunohistochemistry serving as the gold standard. Accurate identification of this tumor is essential for guiding appropriate treatment, which is closely associated with favorable outcomes. Given the potential for unpredictable behavior, long-term clinical follow-up is recommended even for low-grade tumors that initially appear indolent. This case contributes to a broader understanding of the clinicopathological features of SFT in the maxillofacial region.
